# Regulatory mechanisms of the LBD40 transcription factor in *Arabidopsis thaliana* somatic embryogenesis

**DOI:** 10.1002/pld3.547

**Published:** 2023-12-06

**Authors:** Sanjay Joshi, Kristine Hill, Manohar Chakrabarti, Sharyn E. Perry

**Affiliations:** ^1^ Kentucky Tobacco Research and Development Center, 1401 University Dr. University of Kentucky Lexington KY USA; ^2^ Sociology, Philosophy and Anthropology Department University of Exeter Exeter UK; ^3^ School for Integrative Biological and Chemical Sciences University of Texas Rio Grande Valley Edinburg TX USA; ^4^ Dept. of Plant and Soil Sciences, 1405 Veterans Dr., Plant Science Building University of Kentucky Lexington KY USA

**Keywords:** embryogenesis, gene regulation, regeneration, regulatory networks, transcription factor

## Abstract

Somatic embryogenesis (SE) is a process by which an embryo is derived from somatic tissue. Transcription factors (TFs) have been identified that control this process. One such TF that promotes SE is AGAMOUS‐like 15 (AGL15). Prior work has shown that AGL15 can both induce and repress gene expression. One way this type of dual function TF works is via protein interactions, so a yeast 2‐hybrid (Y2H) screen was undertaken. One intriguing protein with which AGL15 interacted in Y2H was LBD40. LBD40 encodes a LATERAL ORGAN BOUNDARIES (LOB)‐domain TF that is unique to plants and is primarily expressed during seed development. Here, we confirm the AGL15‐LBD40 interaction by quantitative assays and *in planta* co‐immunoprecipation. We also document a role for LBD40, and the closely related protein LBD41, in supporting SE. To determine downstream genes potentially controlled by LBD40, chromatin immunoprecipitation followed by high throughput sequencing (ChIP‐seq) was used. More than 400 binding regions for LBD40 were consistently found genome‐wide. To determine genes responsive to LBD40/41 accumulation, RNA‐seq analysis of transcriptomes of wild‐type control and loss‐of‐function *lbd40/lbd41* was performed. Combining these datasets provides insight into genes directly and indirectly controlled by these LOB domain TFs. The gene ontology (GO) enrichment analysis of these regulated genes showed an overrepresentation of biological processes that are associated with SE, further indicating the importance of LBD40 in SE. This work provides insight into SE, a poorly understood, but essential process to generate transgenic plants to meet agricultural demands or test gene function. This manuscript reports on experiments to understand the role that LDB40, a TF, plays in support of SE by investigating genes directly and indirectly controlled by LBD40 and examining physical and genetic interactions with other TFs active in SE. We uncover targets of LBD40 and an interacting TF of the MADS family and investigate targets involvement in SE.

## 1 INTRODUCTION

A valuable method to regenerate plants after transformation or to propagate important genotypes commercially is by producing somatic embryos via a process called somatic embryogenesis (SE). SE provides readily accessible tissues compared with zygotic embryos (ZE), which are embedded in maternal tissues. However, the initial events and gene regulation network in somatic embryo development are still unclear. One of the most studied transcription factors (TFs) that plays a role in the embryonic mode of development and stimulates the production of somatic embryos is *AGAMOUS LIKE15* (*AGL15*, At5g13790) (Harding et al., 2003; Thakare et al., 2008; Zheng & Perry, 2014). AGL15 is a MADS (MCM1, AGAMOUS, DEFICIENS, and SERUM RESPONSE FACTOR) domain TF that accumulates primarily during embryogenesis (Adamczyk et al., 2007; Heck et al., 1995; Perry et al., 1999, 1996; Riechmann & Meyerowitz, 1997). MADS proteins are a family of regulatory proteins in which a 55–60 amino acid domain (the MADS‐domain) is conserved and functions as a DNA‐binding domain. This domain is also involved in dimerization. The cis‐element that MADS‐domain proteins bind is called a CArG motif due to a consensus sequence of C‐A/T‐rich‐G, but there are variations in the particular sequence bound by different MADS‐proteins (Norman et al., 1988; Sommer et al., 1990; Tang & Perry, 2003). AGL15 is part of the sub‐group, which consists of four domains M, I, K, and C. In this group of MADS proteins, there is a short linker called the I‐domain, between the MADS‐domain and a weakly conserved K‐domain that is involved in protein–protein interactions (Riechmann & Meyerowitz, 1997). The function of the C‐terminal domain varies between MADS‐proteins (Egea‐Cortines et al., 1999; Honma & Goto, 2001). The accumulation of AGL15 positively correlates with the production of somatic embryos as discussed further below (Harding, et al., 2003).

*AGL15* and the most closely related Arabidopsis MADS‐box gene *AGL18* encode key regulators of SE, and direct and indirect targets for both TFs have been identified (Joshi et al., 2022; Paul et al., 2022; Zheng et al., 2009). Because AGL15 can directly induce the expression of some genes but repress others, a yeast 2‐hybrid (Y2H) screen was performed to identify proteins that interact with AGL15 and affect the transcription of target genes (Hill et al., 2008). One of the interesting proteins identified in the screen is LBD40 (At1g67100). LBD40 is a TF defined by a conserved N‐terminal Lateral Organ Boundaries (LOB)‐domain, which is specific to plants (Shuai et al., 2002). *LBD* genes in Arabidopsis were first shown as expressed in the base of lateral organs and the proteins they encode have a role in regulating leaf development (Iwakawa et al., 2002; Kong et al., 2017). In Arabidopsis, 43 genes encoding LBD domain proteins have been reported (Shuai et al., 2002).

The Lateral Organ Boundaries (LBD) Domain (also known as ASL for ASYMMETRIC LEAVES2‐LIKE) proteins consists of a conserved cysteine block C‐motif (CX2CX6CX3C), a Gly‐Ala‐Ser (GAS) block, and a leucine‐zipper‐like coiled‐coil motif (LX6LX3LX6L) (Husbands et al., 2007; Majer & Hochholdinger, 2011). The C‐motif block is predicted to be involved in DNA‐binding activity, while the coiled‐coil motif functions in mediating protein–protein interaction. Furthermore, the LBD gene family is divided into two classes according to the structure of the LOB domain (Grimplet et al., 2017; Iwakawa et al., 2002). Class I LBD proteins contain a perfectly conserved CX2CX6CX3C zinc finger‐like domain and a LX6LX3LX6L leucine zipper‐like coiled‐coil motif, whereas class II LBD proteins only have a conserved zinc finger‐like domain (Grimplet et al., 2017; Shuai et al., 2002). *LBD40* is primarily, and perhaps exclusively, expressed during seed development, mainly in the embryo (Zimmermann et al., 2004). LBD40 belongs to class II of the LOB family where it has only the conserved zinc finger‐like motif that is involved in DNA binding (Iwakawa et al., 2002). Various studies on SE transcriptomes have shown *LBD40* expression, like *AGL15,* occurs in all SE systems examined (Braybrook et al., 2006; Suzuki et al., 2007; Wang & Perry, 2013; Zimmermann et al., 2004) and is part of the regulatory network involving other key embryo TFs. Since the master seed regulators LEC1, LEC2, FUS3, and ABI3 directly up‐regulate *LBD40* (Braybrook et al., 2006; Pelletier et al., 2017; Tian et al., 2020; Wang & Perry, 2013; Yamamoto et al., 2010), it is crucial to understand what genes LBD40 in turn regulates. Here, we examine the role of LBD40 in SE, report the targets responsive and bound by LBD40 in embryogenesis, and compare these findings with AGL15. The findings here help to provide a better understanding of gene regulation networks related to SE.

## 2 MATERIALS AND METHODS

### 2.1 Plant material and growth conditions

In this study, Arabidopsis (*Arabidopsis thaliana*) Columbia wild‐type, (Col, wt), loss‐of‐function mutants *lbd40, lbd41, prr5, lea5*, and *agp1* seeds (all Col ecotype) were sown on classic MS media (Murashige & Skoog, 1962) supplemented with 10 g L^−1^ sucrose, .5 g L^−1^ MES, and 7 g L^−1^ agar, pH 5.8. After sterilization, the seeds were chilled at 4C for 3 d and transferred to a growth room with a 16‐h‐light/8‐h‐dark cycle (long‐day light conditions). Ten to 12 days old seedlings were then transferred to a potting mix (ProMix BX; Premier Brands) and grown in a chamber with a 16‐h‐light (20C)/8‐h‐dark (18C) cycle. Shoot Apical Meristem Somatic Embryo(genesis) (SAM SE) was performed as described in the previous report (Harding et al., 2003) and tissue was collected for RT‐PCR and RNA seq at 10 d after the start of culture and flash frozen. Some cultures were grown until 21 dac to score for embryos to verify phenotype. For the developing seeds experiments, flowers were tagged on the day of anthesis, and seeds were collected on 7–8 daf and flash‐frozen in liquid nitrogen for RNA extraction. To generate embryonic culture tissue (ECT) accumulating LBD40, *35S:LBD40‐10x‐c‐myc* transgenic plants were crossed with *35S:AGL15* plants, and the *35S:LBD40‐10x‐c‐Myc:: 35S:AGL15* transgenic lines were recovered by demonstration of hygromycin resistance and characteristic phenotype of AGL15 overexpressors (Wang & Perry, 2013). Developing embryos were cultured as previously described (Harding et al., 2003) to initiate ECT.

### 2.2 Transgene constructs

For the LBD40‐10x‐c‐Myc construct, about 1 kb of the genomic region was amplified and cloned into pENTR/DTOPO vector (Invitrogen). The insert was transferred into the destination vector pGWB19 (10x‐c‐Myc) under the CaMV35S promoter and rbcS terminator, obtained from Dr. T. Nakagawa, Shimane University (Nakagawa et al., 2009) following the manufacturer's instructions for Gateway LR Clonase II Enzyme Mix (Invitrogen, Waltham, MA USA).

### 2.3 Y2H, ChIP and Co‐IP

Please see Hill et al. (2008) for details on the Y2H screen. ECT with 35S:LBD40‐10x‐c‐Myc was fixed in 10 mM potassium phosphate, pH 7, 50 mM NaCl, 0.1 M sucrose, 1% formaldehyde buffer as described previously (Zheng et al., 2009). ChIP was performed as described previously (Joshi et al., 2022). The control was the same tissue, but ChIP was performed without a primary antibody. Co‐IP was done by the protocol described in (Fiil et al., 2008). Briefly, nuclear extraction of *35S:LBD40‐10x‐c‐Myc::35S:AGL15* ECT was done and it was sonicated four times for a 10‐s pulse. After sonication, the nuclear extract is treated with benzonase to remove any nucleic acids. Further, immunoprecipitation using anti‐AGL15 antibody and the Western blot is confirmed using anti‐c‐myc antibody and anti‐AGL15 antibody. The control was the same tissue without immunoprecipitating with anti‐AGL15.

### 2.4 ChIP‐seq and data analysis

Three biological replicates of the ChIP‐seq experiment were performed as described in Paul et al. (2022). To analyze the ChIP‐seq data CLC genomics workbench 12 (Epigenomic Analysis – ChIP‐seq Analysis) was used, following the workflow as described in the CLC Manual using default settings (https://resources.qiagenbioinformatics.com/tutorials/ChIPSEQ_peakshape.pdf). In short, we mapped the reads to the reference (Arabidopsis TAIR10) genome using default setting parameters, Match core = 1, Mismatch cost = 2, Insertion cost = 3, Deletion cost = 3, Length fraction = .5, Similarity fraction = .8, and Ignore the Nonspecific match handling thresholds. The results of read mapping was then used as an input to the TF ChIP‐seq tool to detect significant peaks with Maximum P‐value for peak calling to the value of .05 instead of the default value .1. Thus, we used more stringent threshold. We verified the quality measures for control and samples that were acceptable for the quality threshold. Because we and others have found relevant TF binding sites not only at the 5′ region, but also 3′ and within the gene (Zheng et al., 2009), peaks were associated with both nearby genes, using the Epigenomic analysis tool. The DNA sequences associated with peaks in the ChIP‐seq data were extracted using the Utility tool.

CisGenome was used to extract the DNA binding region information to construct cis‐motif using MEME (Jiang et al., 2010).

### 2.5 RNA isolation and RNA‐seq

Developing seeds (7–8 daf) or SAM SE tissue (10 dac) were collected as described above. RNA was extracted using the QIAGEN RNeasy Plant Mini Kit and supplementing the RLC buffer with 1% final high MW PEG. Two to three biological replicates were prepared and sent for library preparation and RNA‐seq (Novogene, California). Data was analyzed by using CLC genomics workbench 12. The percentage of total mapped with genes for each replicate was determined. The mapped reads, which serve as a measure of sequencing accuracy and lack of contaminating DNA, were in the range of 70–90%. To cast the broadest net, we used *p*‐value <.05 and fold change at least 1.5‐fold cutoffs. Gene ontology (GO) term analysis was performed using PANTHER (Mi et al., 2019) and overrepresented categories using default parameters and FDR < .05 are reported.

### 2.6 ChIP‐qPCR and RT‐qPCR

To confirm the binding of several targets by LBD40 and response to LBD40 accumulation, ChIP‐qPCR and RT‐qPCR, respectively, were performed as described in (Wang & Perry, 2013). For verification of ChIP‐seq targets experiments, ECT expressing *35S:LBD40‐10x‐c‐Myc* was used with anti‐c‐myc and no antibody control (Cell Signaling Technologies, Inc., Danvers, MA, USA). The specific primers used for these experiments are listed in Supplemental Table S1.

### 2.7 Accession numbers

Sequence data from this article can be found in the NCBI SRA database under the Bio project accession numbers PRJNA777254 and PRJNA903892 for AGL18 and AGL15 data, and under the accession number PRJNA994122 forLBD40 data.

## 3 RESULTS

### 3.1 Generation of culture tissue for ChIP (chromatin immuno‐precipitation) and co‐IP experiments

The ECT system has been previously utilized in our lab to characterize genome‐wide binding sites for the embryo development proteins AGL15 (Joshi et al., 2022; Zheng et al., 2009), FUS3 (Wang & Perry, 2013), ABI3 (Tian et al., 2020), and AGL18 (Paul et al., 2022). ECT has been verified and characterized as developing embryo tissue, where the organs develop as cotyledon‐like rather than leafy structures (Harding et al., 2003). Prior studies have shown the active induction of gene expression including those encoding TFs specific to embryogenesis and seed storage genes in ECT (Harding et al., 2003). ChIP performed through ECT is much more robust than from developing seeds, thus, tissue was engineered to map binding sites for LBD40 in ECT. Use of this tissue also allowed comparison with prior work with other TFs in ECT.

The 35S Cauliflower Mosaic Virus (CaMV) promoter was used to drive the expression of *LBD40* with a C‐terminal c‐myc tag (*35S:LBD40‐10x‐c‐myc*), and was placed into plants carrying the *35S:AGL15* transgene that allows stable production of embryonic tissue. Although the tissue contains a *35S:AGL15* transgene, the level of AGL15 protein accumulation in ECT is similar to that in ZE and it appears that consistent, rather than increased accumulation supports embryonic tissue development (Wang & Perry, 2013). Developing embryos from transgenic lines were cultured as described by Harding et al. (2003) and sub‐cultured approximately every 2 to 3 weeks, resulting in stable tissue producing only embryonic organs within a couple of months. Although *LBD40‐c‐myc* is expressed via a *35S* promoter, it is expressed at the same level as the endogenous *LBD40* gene in wild‐type developing seeds suggesting biologically relevant accumulation of this TF (Supplemental Figure S1).

### 3.2 AGL15‐LBD40 protein–protein interaction

**FIGURE 1:**
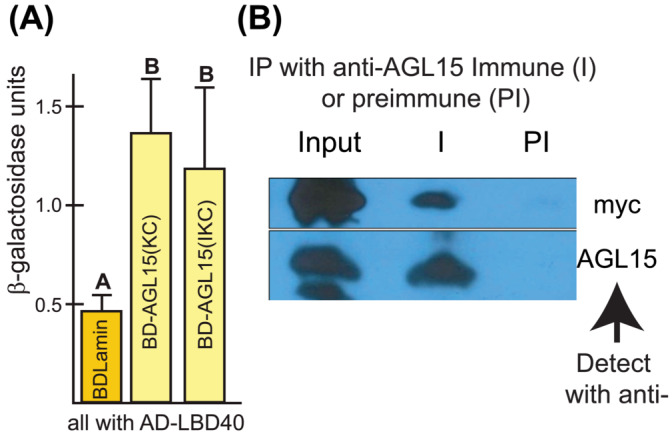
Protein–protein interaction of AGL15 and LBD40. (A) Interaction between AGL15 and LBD40 in yeast. Quantitative CPRG assay shows that LBD40 interacts with AGL15 within the KC domain. The results shown are the average and SD of five separate experiments. Different letters indicate significant difference at *p* < .05. (B) Co‐immunoprecipitation of LBD40‐myc with AGL15. Protein complexes were immunoprecipitated with AGL15 immune serum, or pre‐immune as a control. Detection was first using a c‐myc antibody to detect LBD40‐myc (top panel), followed by AGL15 antibody to confirm good immunoprecipitation (bottom panel).

A yeast 2‐hybrid screen (Y2H) was performed to identify proteins that interact with AGL15, and LBD40 was one of the potential interactors identified. The details of the Y2H screen are provided in Hill et al. (2008). This interaction was further characterized by a quantitative Y2H assay where the interaction of bait and prey led to the induction of a gene encoding β‐galactosidase that can be measured with a quantitative β‐galactosidase assay using chlorophenol red‐β‐galactopyranoside (CPRG). As shown in Figure 1, significantly higher activity is observed when yeast is co‐transformed with AD (activation domain)‐LBD40 and DBD (DNA binding domain)‐AGL15 compared with the control where the DBD construct contained sequences encoding lamin. This is true whether the IKC domains or just the KC domains of AGL15 were used indicating that LBD40 interacts with AGL15 via the K and/or C domains (Figure 1A).

Protein–protein interaction between LBD40 and AGL15 was confirmed *in planta* using co‐immunoprecipitation. Anti‐AGL15 serum was used to immunoprecipitate protein complexes from ECT, the proteins resolved by SDS‐PAGE, immunoblotted, and detection performed for LBD40‐10xc‐myc using a commercial c‐myc antibody as described in Materials and Methods. As shown in Figure 1B, LBD40‐c‐myc was detected when complexes were immunoprecipitated using AGL15 anti‐serum, but not using the pre‐immune control.

### 3.3 LBD40, like AGL15, is involved in SE

**FIGURE 2:**
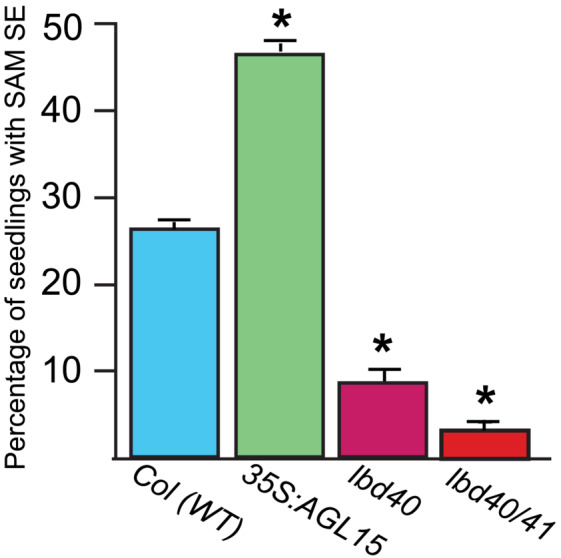
Mutant lines (single *lbd40* and double with *lbd41*) show reduced frequency of SAM SE compared with the control (Col, wt). Different lines of seeds were grown in medium containing 2,4‐D and the seedlings scored for SE development at 21 days after culture (dac). * indicates significant difference at *p* < .05 by a Student's *t*‐test.

Prior work demonstrated that AGL15 and AGL18 accumulation were positively correlated with somatic embryo production. This was true when zygotic explants were cultured, generating ECT, as well as in a SAM SE system. In this later system, when seeds are allowed to complete germination in a liquid medium containing 2, 4‐dichlorophenoxyacetic (2, 4‐D) acid, they will, at some frequency, produce somatic embryo tissue from the shoot apical region (Mordhorst et al., 1998). As verified in the experiments shown in Figure 2, as previously reported, typically Col wt seeds produce callused seedlings where only 20–30% have SAM SE development, *35S:AGL15* shows about twice the frequency of production (i.e., 40–60%). Loss of function, *agl15*, when combined as a double mutant with the closest homolog *agl18*, showed about half the frequency of SAM SE as Col wt (Harding et al., 2003; Thakare et al., 2008). Orthologs of *AGL15* in soybean (Zheng & Perry, 2014) and cotton (Yang et al., 2014) have been found to promote SE when ectopically expressed.

LBD40 interacts with AGL15, and like *AGL15*, is preferentially expressed in developing seeds, in the embryo at similar stages of development as *AGL15* (Zimmermann et al., 2004). In addition, *LBD40*, like *AGL15*, is a direct target of LEC1, LEC2, and FUS3 (Braybrook et al., 2006; Pelletier et al., 2017; Wang & Perry, 2013). Therefore, we tested whether loss‐of‐function of *LBD40* impacted SAM SE development. The loss‐of‐function allele of *lbd40* was combined with a loss‐of‐function in a close homolog *lbd41*, which is also expressed in seeds, although not as preferentially as *LBD40*. LBD41 was also identified as a potential interactor of AGL15 in Y2H but was not pursued further because of failure in follow‐up tests to remove false positives. As shown in Figure 2, Col wt and *35S:AGL15* showed the expected SAM SE production, whereas *lbd40/41* showed a drastic reduction in the ability to produce this tissue. The single *lbd40* mutant also had significant decreased SAM SE compared with Col wt but the single *lbd41* mutant had no significant effect.

### 3.4 Genome‐wide identification of in vivo binding sites for LBD40

To understand how LBD40 may be supporting SE, we determined genes directly and indirectly regulated by this TF. First, we used ChIP‐seq to map binding sites for LBD40. ChIP was performed as in Mi et al. (2020) using an anti‐c‐myc antibody, or no antibody control, co‐precipitated DNA was recovered and sent to BGI Genomics (Cambridge, MA) for library preparation and high throughput sequencing. Three independent experiments were performed using anti‐c‐myc antibody and no antibody (control). CLC Genomics Workbench 12.0 (https://digitalinsights.qiagen.com) was used to analyze the data resulting in 703 target regions (DNA fragments) that were assigned to genes associated with LBD40 (*p*‐value <.05). We used a majority rule to increase number of targets. If at least two of the three replicates shared a binding region, the associated gene was considered as a potential target. This majority rule helps capture important peaks that would be missed because of low reads or high noise background in some replications (Yang et al., 2014). Out of 703 targets, 424 common binding regions were found in three biological replications (therefore an additional 279 were in at least two out of three replications). A comprehensive list is provided in Supplemental Dataset S1.

We further analyzed the list of genes with potential regulatory regions associated with LBD40 using the GO term enrichment tool by Protein Analysis Through Evolutionary Relationships (PANTHER) (Mi et al., 2019) and found many categories were overrepresented, including “regulation of post‐embryonic development” (GO:0048580); 3.29‐Fold Enrichment (FE); False Discovery Rate (FDR) 8.70E‐03) and “response to osmotic stress” (GO:0006970); 2.46 FE; FDR 4.63E‐02). Other selected categories within “biological processes” are shown in Table 1.

**TABLE 1:** GO enrichment analysis for genes (703) with regulatory regions associated with LBD40. PANTHER classification system was used to discover significantly (FDR < .05) overrepresented. Fold enrichment (FE) compares the dataset with the whole Arabidopsis genome. Release 2021‐11‐16.

GO categories for LBD40 bound genes enriched	Fold enrichment	FDR
Regulation of post‐embryonic development (GO:0048580)	3.29	8.70E‐03
Flower development (GO:0009908)	3.07	1.87E‐02
Regulation of reproductive process (GO:2000241)	2.89	2.86E‐02
Response to osmotic stress (GO:0006970)	2.46	4.63E‐02
Response to abiotic stimulus (GO:0009628)	1.98	9.23E‐04
Regulation of gene expression (GO:0010468)	1.64	2.15E‐02

Abbreviations: FDR, false discovery rate; FE, fold enrichment; GO, gene ontology.

**FIGURE 3:**
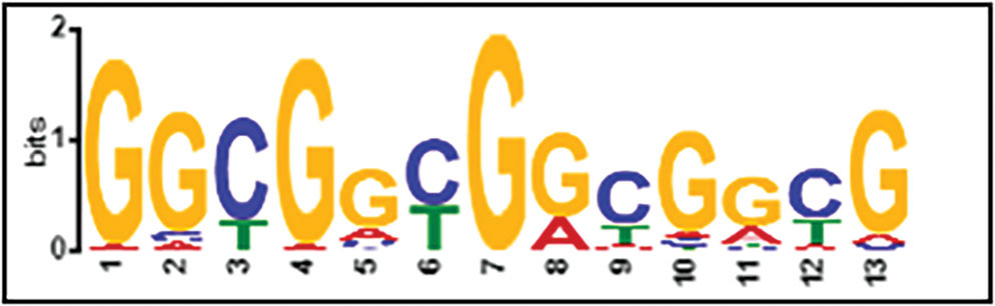
ChIP‐MEME analysis for 424 common binding regions found in three biological replicates of the ChIP experiment. The sequences of regions identified as bound by LBD40 were retrieved using CisGenome (Jiang et al., 2010) and ChIP‐MEME performed using default settings as in Macanick and Bailey (2011).

Sequences corresponding to the peaks of binding were obtained, and the average length was 150 bp. Locations of bound sites were obtained using CisGenome. As shown in Table 2, most of the sites were intragenic including in exon regions. Furthermore, we obtained sequences corresponding to the bound regions found in all three biological replicates of ChIP‐seq and used ChIP‐MEME to assess whether any motifs were significantly overrepresented. As shown in Figure 3, a GC‐rich region was found to be overrepresented. This motif resembles a cis motif bound by the members of APETALA2/ETHYLENE RESPONSIVE FACTOR (AP2/ERF) family (Shoji et al., 2013), and is related to a site bound by LOB, the founding member of the LOB‐domain family (Husbands et al., 2007).

**TABLE 2:** Locations of sequences of total bound sites and common sites in three replications immunoprecipitated with antibody to isolate LBD40‐c‐myc complexes. Sequences were obtained using CisGenome and mapped using The Arabidopsis Information Resource 9 to intergenic and intragenic regions. Totals are more than 100% because some sequences span more than one region.

Region of gene	Location of total bound sites (703 sites)	Location of bound sites common 424 sites
Intergenic	145 (20%)	97 (23%)
Intragenic	557 (79%)	327 (77%)
Exon	532 (75%)	310 (73%)
Intron	31 (4%)	21 (5%)
CDS	465 (66%)	276 (65%)
UTR	73 (10%)	40 (9%)
5′UTR	27 (3%)	11 (3%)
3′UTR	47 (6%)	29 (7%)
Intergenic (≤1 kb‐from‐gene)	312 (44%)	200 (47%)
TSSup1k	175 (24%)	110 (26%)
TESdown1k	175 (24%)	107 (25%)
Intergenic (≤10 kb‐from‐gene)	702	423
TSSup10k	680 (96%)	409 (96%)
TESdown10k	670 (95%)	401 (95%)
Intergenic (≤100 kb‐from‐gene)	702	424
TSSup100k	702	424
TESdown100k	702	424

Abbreviations: CDS, coding sequences; TES, transcriptional end site; TSS, transcriptional start site; up/down 1 kb, within 1 kb of the TSS and/or TES as indicated; UTR, untranslated region of the transcript 5′ and 3′ of the CDS that will be translated to protein.

### 3.5 Validation of selected targets of LBD40‐DNA interactions

**FIGURE 4:**
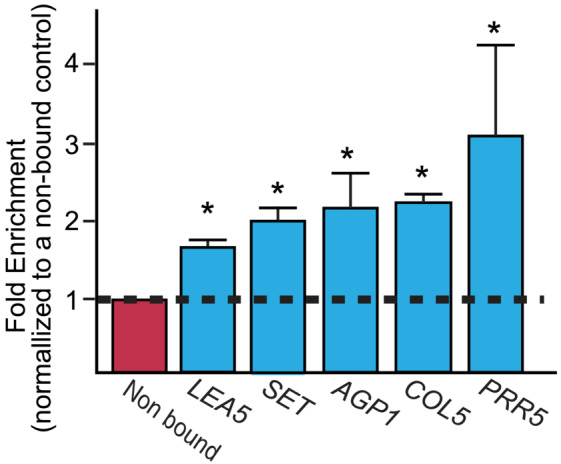
Fold enrichment measures the amount of the suspected target fragment recovered in the LBD40‐10x‐cmyc ChIP experiment using anti‐c‐myc antibody compared with a no antibody control. Data was normalized to a region that is not bound by LBD40. Data shown are the average and SE of four biological replicates. * represents significance of *p* < .05.

Among the LBD40 associated 703 targets, we focused on genes that were likely to have a function in seed development, embryogenesis, or stress response. To confirm the association of LBD40 with genomic regions of interest, ChIP‐qPCR was performed on biologically independent ChIP experiments. Quantitative PCR was used to assess the amount of DNA fragment recovered by ChIP using at least four biological replicates of the ChIP experiment that were independent of those used for ChIP‐seq. The “fold enrichment” is the amount of target recovered from the LBD40‐c‐myc tissue compared with the negative control (no antibody) and normalized to a non‐bound NB1 (Supplemental Table S1) fragment we have used in other studies. This NB1 appeared to be appropriate for LBD40, as it was not associated with LBD40 in the ChIP‐seq experiments. As shown in Figure 4, the targets studied were AT4G02380‐*SENESCENCE‐ASSOCIATED GENE 21 (LEA5)* (Candat et al., 2014; Salleh et al., 2012), AT1G01920‐*SET DOMAIN‐CONTAINING PROTEIN (SET)* (Xu et al., 2012; Zhou et al., 2020), AT5G64310‐*ARABINOGALACTAN PROTEIN 1 (AGP1)* (Pereira et al., 2014; van Hengel et al., 2001), AT5G57660‐*CONSTANS‐LIKE 5 (COL5)* (Gomez et al., 2018),and AT5G24470‐*PSEUDO‐RESPONSE REGULATOR 5 (PRR5)* (Yang et al., 2021). All of the selected targets tested showed significant fold‐ enrichment relative to the control immunoprecipitation, supporting the ChIP‐seq data that all of the targets were associated with LBD40 (Figure 4).

### 3.6 Transcriptome in response to *lbd40/41* in SAM SE

RNA‐seq was used to assess gene expression in response to LBD40 by comparing the transcriptome in the *lbd40/41* mutant with Col wt. The *lbd40/41* double mutant was used because it consistently showed a large reduction in SE production in the SAM SE system, indicating redundancy. In addition, Y2H indicated that AGL15 also may interact with LBD41. Studies have indicated binding could be possible without any significance at the gene expression level (Lee et al., 2007; Oh et al., 2009; Wyrick & Young, 2002), so we wanted to evaluate the genes that respond to changes of LBD40/41. For this purpose, we used two different tissues: (1) 10‐days‐after‐culture (dac) before any obvious embryo development was apparent from SAM SE system and, (2) developing seeds containing ZE harvested from siliques 7–8 days after flowering (daf). Two biological replicates were performed for each genotype.

For the SAM SE system, we found a total of 2,426 genes showed significantly increased transcript accumulation (i.e., repressed targets; *lbd40/41*:Col ≥ 1.5‐fold) whereas 1,145 genes showed decreased transcript accumulation (i.e., induced targets; *lbd40/41*:Col ≤ .66‐fold) compared with control (Col wt). A list of genes is provided in Supplemental Dataset S2. For developing seeds, using the same cutoffs as SAM SE, there were approximately 2,871 with altered transcript accumulation. The result showed a total of 785 genes that are upregulated in response to the loss of LBD40/41 (i.e., repressed targets), and 2,086 genes are downregulated (i.e., induced targets; Supplemental Dataset S3).

**FIGURE 5:**
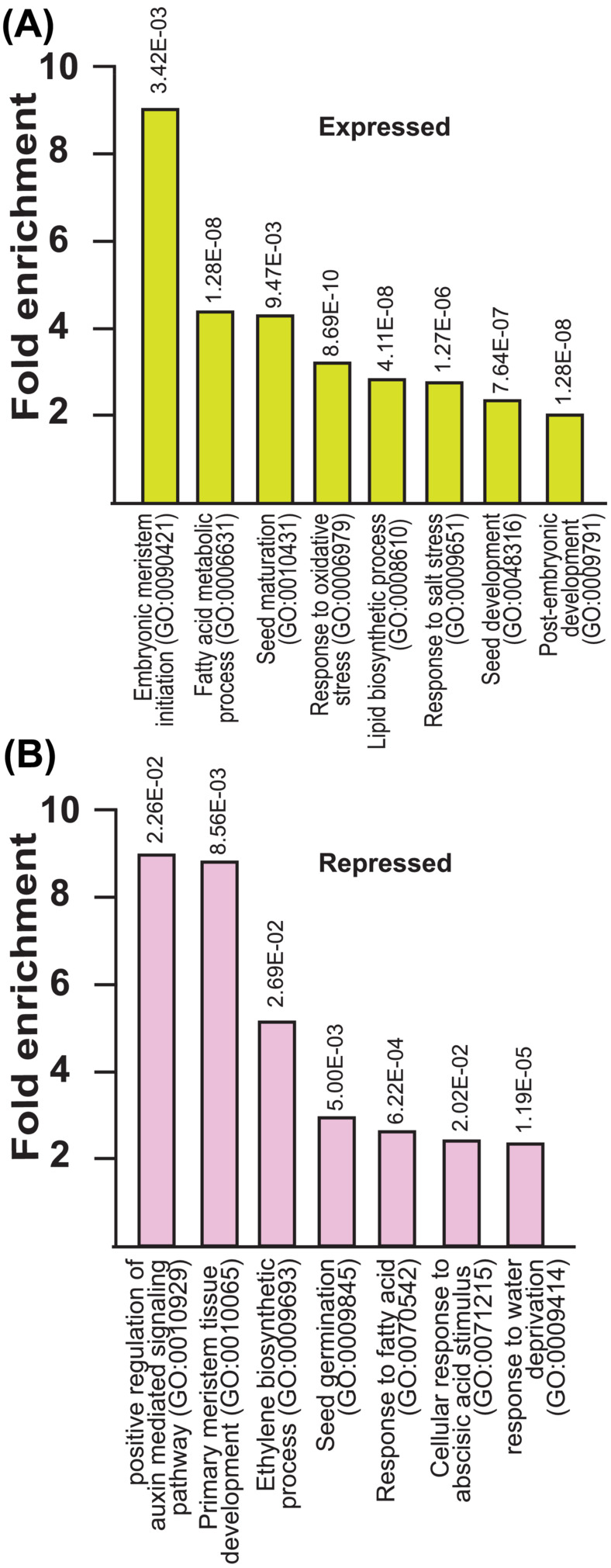
Functional characterization of genes responsive to LBD40/41 accumulation. A. Induced, and B. Repressed list of gene expression in RNA‐seq analysis of SAM SE comparing *lbd40/41* to Col, wt using the PANTHER classification system statistical overrepresentation test with default settings (FDR < .05). Select GO biological processes (release data 2021‐11‐16) are shown as fold enrichment in the responsive list compared with the entire Arabidopsis genome.

We further analyzed the differentially expressed genes from the RNA‐seq using the GO term enrichment tool for both SAM SE and seed gene lists. In the case of SAM SE, the induced gene list contained many categories that were overrepresented, including “embryonic meristem initiation” (GO:0090421); 9.01 FE; FDR 3.42E‐03 and “response to oxidative stress” (GO:0006979); 3.21 FE; FDR 6.69E‐10. For repressed genes in the context of SAM SE, we found enrichment of genes involved in “positive regulation of auxin‐mediated signaling pathway (GO:0010929); 8.94 FE; FDR 2.26E‐02, “ethylene biosynthetic process” (GO:0009693); 5.12 FE; FDR 2.69E‐02). Other selected categories within “biological processes” for both induced and repressed genes are shown in Figure 5. We also performed a similar GO term enrichment analysis in gene expression analysis of *lbd40/41* seeds (7–8 daf). We found an interesting overrepresentation of biological processes including “negative regulation of cell death” (GO:0060548); 5.11 FE, FDR 1.53E‐03, “positive regulation of abscisic acid‐activated signaling pathway” (GO:0009789); 3.33 FE, FDR 3.53E‐02) for induced genes. The detailed categories within “biological processes” for both induced and repressed genes for seeds are shown in Table 3.

**TABLE 3:** GO enrichment analysis for transcripts which are upregulated (repressed) and downregulated (induced) in RNA‐seq *lbd40/41 seeds* (7–8 daf) compared with Col wt. PANTHER classification system was used to discover significantly (FDR < .05) overrepresented. FE compares the dataset with the whole Arabidopsis genome. Release 2021‐11‐16.

GO categories for seeds repressed genes enriched for:	Fold enrichment	FDR
Cellular response to abscisic acid stimulus (GO:0071215)	3.86	4.34E‐02
Chromatin organization (GO:0006325)	3.5	1.25E‐02
Gene expression (GO:0010467)	2.21	6.82E‐07
Seed development (GO:0048316)	2.09	3.43E‐02

GO categories for seeds induced genes enriched for:	Fold enrichment	FDR
Photosynthesis, light harvesting in photosystem I (GO:0009768)	9.55	2.00E‐06
Negative regulation of cell death (GO:0060548)	5.11	1.53E‐03
Positive regulation of signal transduction (GO:0009967)	3.34	1.11E‐03
Positive regulation of abscisic acid‐activated signaling pathway (GO:0009789)	3.33	3.53E‐02
Seed germination (GO:0009845)	3.03	4.25E‐03
Response to fatty acid (GO:0070542)	2.96	4.58E‐05
Response to oxidative stress (GO:0006979)	2.77	2.19E‐10
Lipid localization (GO:0010876)	2.57	3.18E‐03

Abbreviations: FDR, false discovery rate; FE, fold enrichment; GO, gene ontology.

### 3.7 Verification of the response of target genes to decrease‐of‐function as found in *lbd40/41*

**FIGURE 6:**
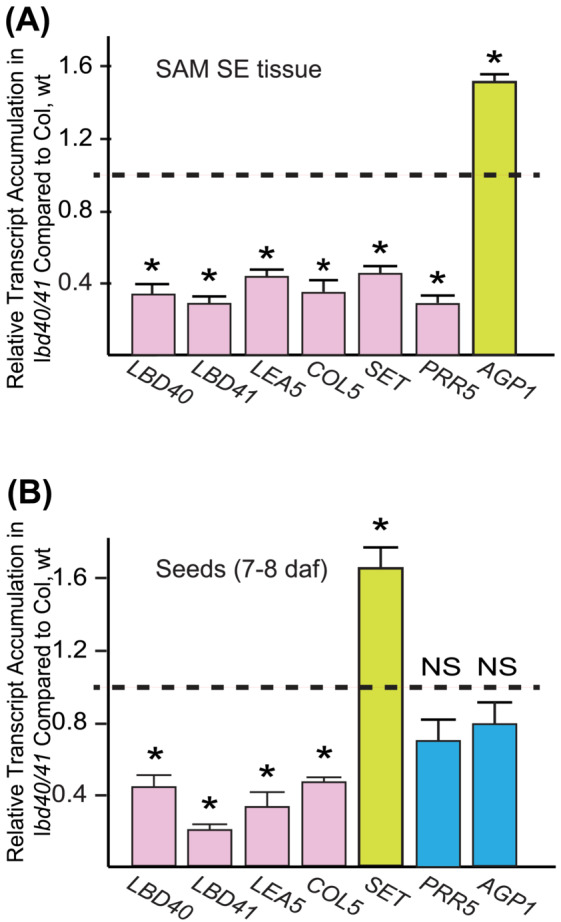
Response of selected putative targets to *lbd40/41*. qRT‐PCR was used to assess transcript accumulation in (A) 10 dac SAM SE tissue and (B) 7–8 daf developing seed in the *lbd40/41* knock‐down mutant compared to Col, wt. Data shown are the average and SE of three biological replicates. * represents significance at *p* < .01 as determined using a Student's *t*‐test. NS, not significant.

To verify the RNA‐seq data, we validated a few potential genes involved in embryogenesis. We tested if these genes were responsive to the accumulation of LBD40 using reverse transcription‐qPCR (RT‐qPCR). We studied transcript accumulation in *lbd40/41* 10 dac SAM SE culture tissues and staged 7 to 8 daf seeds with Col wt SAM SE tissues and seeds, respectively. As expected, *LBD40* and *LBD41* had decreased transcript abundance in the mutant compared with Col, but neither were complete knock‐out mutants. In addition, *COL5, and LEA5* showed significantly reduced levels of transcript accumulation compared with the Col wt in both seeds and SAM SE tissues for the mutant line supporting the model that LBD40/41 induces expression, as measured by transcript accumulation, of these genes in both tissue contexts (Figure 6). The transcript from the *SET* identified as a direct target, showed reduced transcript in *lbd40/41* seeds compared with Col wt, but increased transcript abundance in SAM SE (Figure 6). On the other hand, *AGP1* was induced in SAM SE tissue (reflected by a decrease in transcript in the *lbd40/41* compared with Col wt), but there was no significant change in seeds (Figure 6). These qRT‐PCR results agree with the RNA‐seq data for the different genes and tissues.

### 3.8 Identification of putative direct and indirect targets of LBD40

**FIGURE 7:**
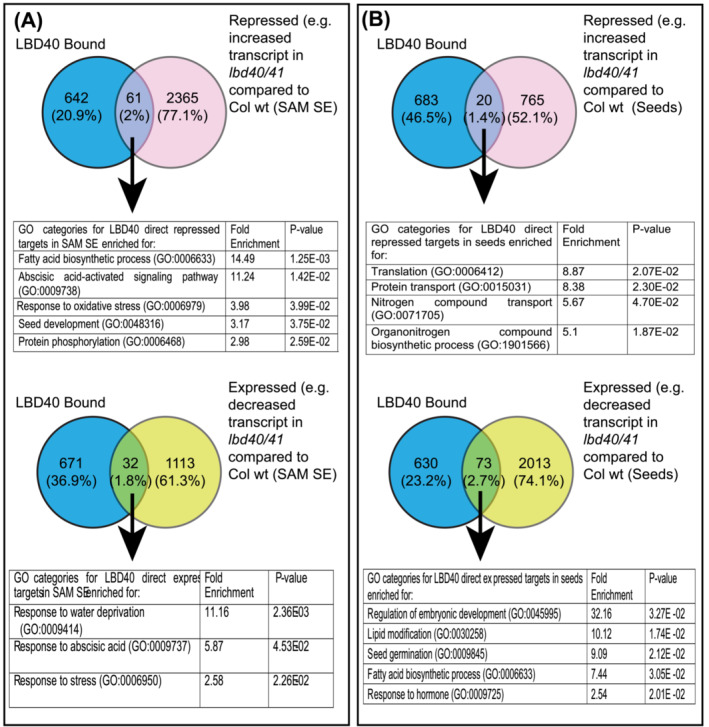
Overrepresented GO categories for genes associated with LBD40 by ChIP‐SEQ and responsive to loss‐of‐function lbd40/41 in 10 dac SAM SE (A) or 7–8 daf developing seeds (B). Panther classification system (release 2021‐11‐16) was used to find the significantly (*p*‐value <.05) overrepresented categories of genes. Here, fold‐enrichment compared the direct responsive populations with the Arabidopsis genome. The number of genes bound by LBD40 and/or responsive or both are shown as well as the percentage of the total of bound and/or responsive in the Venn diagram. A. Direct expressed and repressed in response to *lbd40/41* in SAM SE. B. Direct expressed and repressed in response to *lbd40/41* in developing seeds.

The potential direct and indirect targets of LBD40 were identified by examining the list of genes between ChIP‐seq and RNA‐seq. The genes with potential regulatory regions isolated by LBD40 ChIP and that respond to LBD40/41 accumulation represent direct, responsive targets (Supplemental Dataset S4). Meanwhile, those not associated with LBD40, but that show response to LBD40/41 accumulation are potentially indirect, responsive targets. Some targets are directly bound but do not show obvious perturbations in transcript accumulation under the conditions we tested and the cutoffs we used. As shown in Figure 7 a total of 93 genes were found to be direct and responsive targets, among which 61 (2%) appeared to be directly repressed by LBD40 in SAM SE tissue. On the other hand, 32 genes of the 1,145 (1.8%) were directly induced targets. Further, we examined these lists for overrepresentation of GO terms. Directly induced genes were overrepresented in categories including response to abscisic acid (GO:0009737), and response to stress (GO:0006950). Directly repressed genes were overrepresented in categories including fatty acid biosynthetic process (GO:0006633), and seed development (GO:0048316) as shown in Figure 7A.

Similarly, 73 genes of the 2086 (2.7%) induced targets, and 20 genes of 785 (1.4%) repressed targets were also directly associated with LBD40 in Arabidopsis seeds (Figure 7B). GO terms for overrepresented categories were studied in these lists (Supplemental Dataset S4). The genes involved in the regulation of embryonic development (GO:0045995), and response to hormones (GO:0009725) were overrepresented in the directly induced genes. Directly repressed genes were overrepresented in developmental processes (Figure 7B). The lists of genes for both direct induced and direct repressed targets of LBD40 from the SAM SE and seed studies are in Supplemental Dataset S4. Genes that respond to LBD40 accumulation in both developing seeds and SAM SE are shown in Supplemental Dataset S5. While some direct and indirect targets of LBD40 responded congruently to this TF in both contexts, others showed opposite response, likely reflecting the context.

### 3.9 Genes with regulatory regions bound by both LBD40 and AGL15

**FIGURE 8:**
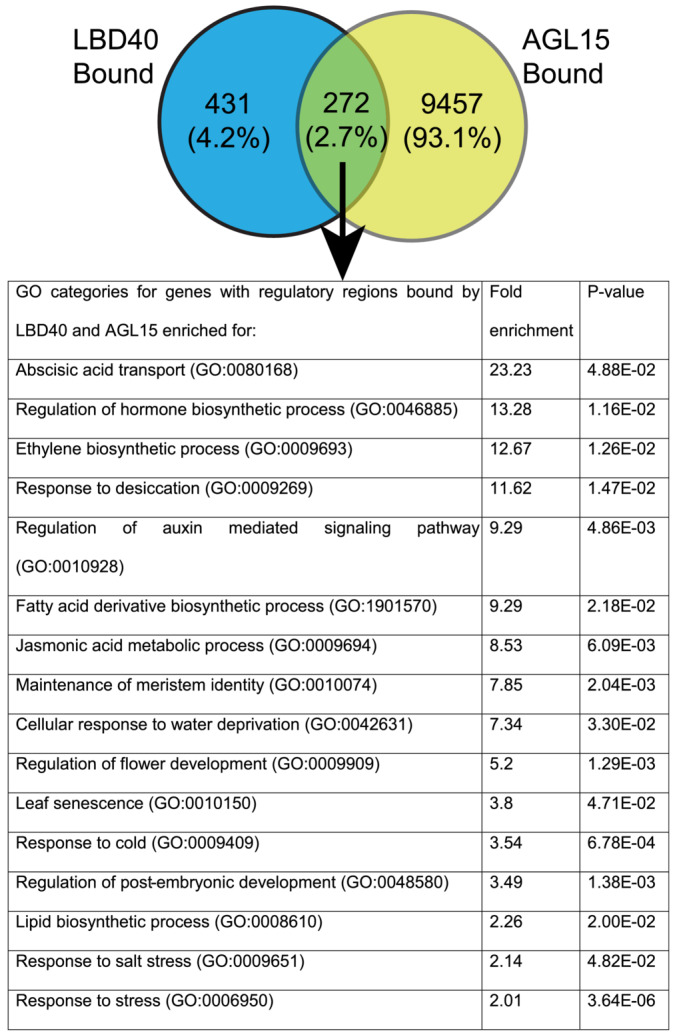
GO enrichment analysis for genes with regulatory regions associated with LBD40 and AGL15. Panther classification was used to find the significantly (*p* < .05) overrepresented categories of genes. Fold enrichment compares the dataset of co‐bound genes to the Arabidopsis genome (GO release 2021‐11‐16). The Venn diagram shows the number of genes bound by LBD40 and/or AGL15.

Since AGL15 and LBD40 proteins interact with each other, we analyzed the common bound regions between both TFs. We utilized the ChIP‐seq data results from *35S:AGL15* ECT (Paul et al., 2022) to identify the common targets. Paul et al. (2022) reported 9,729 potential bound targets for AGL15 and there were 272 genes (2.7%) bound by both LBD40 and AGL15 (Figure 8). The list of genes that are bound by both TFs is in (Supplementary Dataset S6). The biological functional categories were analyzed for these common targets using PANTHER GO, and the results showed genes involved in developmental processes such as meristem identity (maintenance of meristem identity (GO:0010074), hormone regulation involving auxin, ethylene, abscisic acid, and SE‐related responses such as response to stress (GO:0006950), and response to desiccation (GO:0009269) (Figure 8).

The list of directly induced and repressed LBD40 targets in Supplemental Dataset S4 were compared with sites documented as bound by AGL15 by ChIP‐chip and/or ChIP‐seq and for which transcriptome data includes information supportive of expression or repression. This is summarized in Supplemental Tables S2 and S3.

### 3.10 Downstream targets of LBD40 impact on SAM SE

**FIGURE 9:**
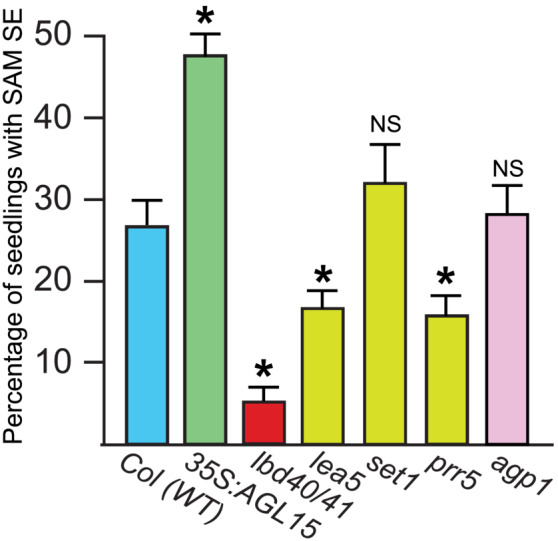
SAM SE production by select direct expressed (yellow) and repressed (pink) targets of LBD40/41. Data shown are the average and SE of at least two biological replicates. *Represents significance at *p* < .01 as determined using a Student's *t*‐test. NS, not significant.

We selected several targets of interest of LBD40/41 to test if they have biological significance for SE. These targets included *AGP1* for which data from studies on AGL15 support a role in direct repression, and *PRR5* that is a potential directly induced AGL15‐LBD40 target. As shown in Figure 9, several targets determined to be directly expressed by LBD40/41 are involved in producing SAM SE, in that mutants show decreased SAM SE as expected. Others, including SET and AGP1 had diverse response comparing SAM SE with seeds and did not show a significant change in production of SAM SE.

## 4 DISCUSSION

Prior work documented that AGL15, a MADS‐factor, can promote SE in a variety of dicots (Harding et al., 2003; Yang et al., 2014; Zheng & Perry, 2014), and expression of orthologs in other species correlate with embryo development (Joshi et al., 2022). AGL15 is a direct induced target of several key embryo identity TFs including LEC1, LEC2, FUS3, but not ABI3 (the so‐called LAFL factors) (Braybrook et al., 2006; Pelletier et al., 2017; Tian et al., 2020; Wang & Perry, 2013). These LAFL TFs are also able to induce embryonic programs in tissues after completion of germination to different extents (Gazzarrini et al., 2004; Lotan et al., 1998; Stone et al., 2001). Direct and indirect targets of AGL15 were determined to investigate the mechanism of SE promotion, revealing that AGL15 can directly induce as well as repress gene expression (Joshi et al., 2022; Zheng et al., 2009). To better understand how this occurs Y2H studies were undertaken revealing that AGL15 includes a motif (LxLxL) that can interact with proteins such as TOPLESS (TPL) and SIN3 ASSOCIATED POLYPEPTIDE P18 (SAP18) that recruit histone deacetylase complexes (HDAC's) (Causier et al., 2012; Hill et al., 2008). The LxLxL motif was found to be necessary to interact with SAP18, but was not essential for promoting SAM SE (Joshi et al., 2021). However, conversion of AGL15 by addition of a strong transcriptional activation domain (VP16) was detrimental to SAM SE production demonstrating a needed balance of gene regulation (Joshi et al., 2021). Histone deacetylation is generally, but not exclusively, correlated with repression of gene expression (Liu et al., 2014).

While the results discussed above explain how AGL15 may repress gene expression, AGL15 does not contain any clear transcriptional activation domain, such as acidic‐rich or glutamine and/or proline rich domains and does not autoactivate in yeast (Hill et al., 2008).. Therefore, we further investigated other proteins uncovered by Y2H to interact with AGL15 and focus here on one such interactor, LBD40. We chose this potential protein interactor because regardless of whether we could confirm *in planta* interaction with AGL15 (that we did [Figure 1]), given other considerations (outlined below), as well as the fact that it appears necessary for SAM SE development (Figure 2), information on this gene product is important to understanding SE. Thus we proceeded with identifying targets of this TF.

LBD40, like AGL15, is (1.) a directly induced target of the LAFL TFs, in this case including ABI3 (Pelletier et al., 2017; Stone et al., 2001; Tian et al., 2020; Wang & Perry, 2013; Yamamoto et al., 2010); (2.) preferentially expressed in embryos at similar stages as *AGL15* (Zimmermann et al., 2004); (3.) is necessary for efficient SE (Figure 2) and (4.) as shown here, has a documented interaction with AGL15 in Y2H and *in planta* (Figure 1). Also, it is intriguing, based on data from Sugimoto et al. (2010) and Che et al., (2006) respectively, available in eBAR, that LBD40 and AGL15 are induced during dedifferentiation of tissue on Callus Inducing Media (Sugimoto et al., 2010). LBD40 is a plant‐specific TF that includes a region with many acidic residues in the C‐terminal region (20% of the C‐terminal 83 amino acid residues are acidic), a hallmark of some transcriptional activation domains (Roberts, 2000). Therefore, a question was whether co‐regulation by AGL15 and LBD40 would induce gene expression. Double overexpressors of AGL15 and LBD40 were very weak and did not set seed well.

As shown in Figure 2, the *lbd40/41* double mutant showed very consistent and dramatic decreased ability to form SAM SE. The single *lbd40* decreased function mutant (not a knock‐out, but rather a knock‐down) also consistently showed a significant decrease in SAM SE. Why do we look at the double mutant with *lbd41* (again a knock‐down but not a knock‐out)? First, LBD41, the closest related protein to LBD40, was also identified as an interactor of AGL15 in Y2H but was not pursued as it was able to autoactivate. LBD41 is the closest homolog to LBD40 and while expressed in the seed, it shows a broader expression pattern (Zimmermann et al., 2004). The knock‐down of *lbd41* did not have a significant impact on SAM SE, even though it enhanced loss‐of‐function of *lbd40* for this phenotype. This is similar to the situation with AGL15, and its closest related protein AGL18: the double mutant *agl15 agl18* has a larger, and more consistent impact on SAM SE than single mutants (Thakare et al., 2008). While overexpression of *LBD40* showed enhancement of SAM SE, this result was not completely reproducible. Thus, the *lbd40/41* mutant was compared with Col wt for the transcriptome analysis.

Given all of the above, the gene regulatory network of LBD40 in supporting SE was of particular interest. To that end, we assessed genome‐wide binding by LBD40 and transcript accumulation in response to accumulation of LBD40/41 using ChIP‐seq and RNA‐seq respectively. The GO term of genome‐wide binding of LBD40 targets showed response to stress and regulation of gene expression (Table 1) as also observed in AGL15 and AGL18 regulation (Paul et al., 2022), supporting a mechanism of LBD40 involvement in SE as stress is a major triggering factor for SE. LOB domain proteins bind DNA in a sequence‐specific manner via GCGGCG consensus motifs (Husbands et al., 2007), and cis‐motif analysis for LBD40 binding targets common in three replications showed a GC rich motif similar to the this sites as well as sites recognized by the ethylene response factor (ERF) family (Figure 3).

We further conducted a transcript accumulation study for *lbd40/41* compared with Col (wt) for 10 dac SAM SE and 7–8 daf seeds as we were curious as to overlap in these two contexts. While some genes are shared in response to LBD40 accumulation, others are not (Supplemental Dataset S6), likely reflecting context and/or particular stage of development within the context studied. Interestingly, GO term enrichment study of both induced and repressed genes on both studies helps us to provide better insights into the biological function of LBD40. Our analysis showed LBD40 involvement in embryonic meristem initiation, stress, and hormone (Figure 5). Recently, there have been more studies highlighting the diverse functions of *LBD* genes in many aspects of plant development, including root, leaf, inflorescence, pollen development, embryo development, plant regeneration, photomorphogenesis, pathogen response, and anthocyanin and nitrogen metabolism (Grimplet et al., 2017; Kong et al., 2017; Xu et al., 2016; Zhang et al., 2020). For example, studies have shown in Arabidopsis that lateral root formation is regulated via *LBD16, LBD29*, and *LBD18*, targeting AUXIN RESPONSE FACTORs (ARFs) ARF7 and ARF19, suggesting *LBD* genes involved in auxin signaling cascades (Inukai et al., 2005; Okushima et al., 2007).

We next analyzed direct targets that respond to LBD40 accumulation. We combined transcriptomic information with the ChIP‐seq data to identify the direct targets and indirect responsive targets. We found a fraction of LBD40‐associated genes that showed response to LBD40 (about 2%) under the conditions tested and cutoffs used (Figure 7). This percent was lower than the general trend of several other TFs or particular conditions under which response is measured. For instance, in AGL18 less than 10% were shown to be involved as direct target compared with control (Paul et al., 2022); similarly, only a portion of AGL15‐associated genes showed response to AGL15 (Zheng et al., 2009) as well as in other studies (Tian et al., 2020; Wang & Perry, 2013). As explained in Paul et al. (2022), one of the possible reasons for this difference between binding and response could be due to conditions where particular cofactors or signals are required to activate a response.

However, the direct targets show that LBD40/41 can act as a transcriptional repressor as well as activator. Also, while more than 200 genes were directly targeted by AGL15 and LBD40 (Figure 8), few showed response to both factors (Supplemental Tables S2 and S3), but those that did include co‐regulated induced genes (PRR5) as well as co‐regulated repressed genes (ABI4 and AGP1), thus LBD40 does not appear to contribute transcriptional activity specifically to gene expression for AGL15. While LBD40 has enrichment of acidic residues in the C‐terminal end (20% as discussed above), this enrichment is not as high as found for VP16, a recognized strong transcriptional activation domain (Sadowski et al., 1988) (28.7% of the 80 residues in the activation domain are acidic). Also, it is notable that both LBD40 and LBD41 have LxLxL motifs that could be involved in recruitment of components of histone deacetylase complexes and may thereby repress gene expression. While LBD40 does not as yet have a documented interaction with SAP18, TPL or the TPL related protein, (TPR), LBD41 does interact with TPL and one of the TPRs (as does AGL15) based on information in BioGrid (Oughtred et al., 2019). It should be noted that AGL15 does interact with other proteins including SEP3 and AP1 with documented transcriptional activation domains (de Folter et al., 2005; Hill et al., 2008; Honma & Goto, 2001; Oughtred et al., 2019). Both SEP3 and AP1 also have LxLxL motifs. Thus, much remains to be understood in the complex interactions or proteins to form complexes, as well as interactions with DNA.

### 4.1 Stress could induce SE via LBD40

When we analyzed the GO term for the direct targets of LBD40 from SAM SE tissue “abscisic acid signaling pathway” and “response to stress” were the main biological functions (Figure 7). We chose the various targets involved in these processes from the list in Supplementary Dataset S4. Selected targets included *SENESCENCE‐ASSOCIATED GENE 21 (LEA5/SAG21), ABA INSENSITIVE 4 (ABI4),* and *PSEUDO‐RESPONSE REGULATOR 5 (PRR5)*. LBD40. as well as AGL15, directly expresses *PRR5* (this paper and datasets from (Zheng et al., 2009). PRR5 is shown to interact with ABI5 to regulate ABA during seed germination (Huang et al., 2017; Shi et al., 2021; Yang et al., 2021). PRR5 is one of the circadian clock proteins and positively modulates ABA signaling. Overexpression of *PRR5* inhibits seed germination (Yang et al., 2021). Similarly, *SAG21/LEA5* expression responds to hormones (ABA) and abiotic stress (drought, salinity, and cold) (Salleh et al., 2012). Several types of stress play a vital role in SE. Studies have shown SE induction of genes involved in stress (for review, see (Elhiti & Stasolla, 2022; Méndez‐Hernández et al., 2019; Nowak & Gaj, 2016). One of the well‐studied and common types of stress is caused by 2,4‐D (a synthetic auxin) which leads to induction of embryo in somatic cells (Li et al., 2021). Several TFs that can induce SE respond either directly or indirectly to auxin including BBM, FUS3, LEC1, LEC2, WUS, and AGL15 (reviewed in (Tian et al., 2020). Studies have shown treating cells with ABA and polyethylene glycol (PEG) has supported regeneration of somatic embryos and maturation (Stasolla et al., 2003).

**FIGURE 10:**
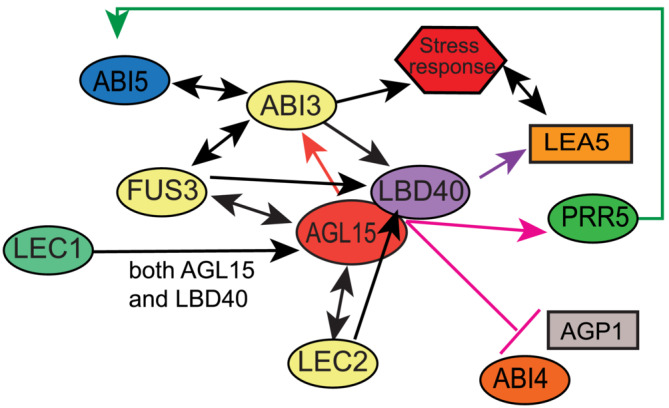
Working model showing interactions between major factors that influence SE. AGL15 and LBD40 show protein–protein interaction. TFs are in ovals; B3 domain in yellow, CCAAT in aqua, LOB in purple, MADS in red, PRR in green, ERF/AP2 in orange, and bZIP in blue. Pink arrow or bar indicate genes directly induced or repressed by AGL15 and LBD40. Not all interesting interactions are shown in the interest of clarity. Please see the Discussion for relevant references.

Comparing LBD40 with AGL15 ChIP‐seq showed around 40% of targets associated with LBD40 were also present as associated with AGL15. Further, The GO term for the regulatory regions associated with LBD40 and AGL15 showed gene potentially involved in SE production such as categories “regulation of hormone”, “response to desiccation”, “maintenance of meristem identity” supportive with prior studies (Mi et al., 2020; Tian et al., 2020). Additionally, we compared the targets of AGL15 with LBD40 and found shared repression targets such as ABI4, and AGP1. Arabinogalactan proteins (AGP) are highly glycosylated and structurally complex proteins that have a role in stimulating SE (Majewska‐Sawka & Nothnagel, 2000). *AGP1* showed direct repression in response to both AGL15 and LBD40 accumulation. SAM SE experiments for *AGP1* mutant line showed somatic embryos similar to Col (wt.) (Figure 9), which could be due to redundancy of AGPs. However, more experiments are needed to confirm the phenotype. Interestingly, *AGP1* is shown to be induced in female reproductive tissue (Pereira et al., 2014; van Hengel et al., 2001). Some of the AGPs, a subgroup of fasciclin‐like AGPs, (FLAs) are present in non‐embryogenic tissue and playing a role as a nursing function to the cells that actually form the SEs (Majewska‐Sawka & Nothnagel, 2000). Based on our findings, we propose a model (Figure 10) demonstrating the transcriptional regulation by LBD40 that might support SE, with protein–protein interaction of LBD40 and AGL15.

## AUTHOR CONTRIBUTIONS

Conceptualization, S.P. S.J. and K.H..; Methodology, S. J and K.H.; Validation, S.J. and K.H.; Formal analysis, S.J., M.C. and S.P.; Investigation, S.J., K.H., M.C. and S.P.; Data curation, S.J.; Writing, original draft preparation, S.J.; Writing, review and editing, S.J., K.H., M.C. and S.P.; S.P.; Project administration, S.P.; Funding acquisition, S.P. All authors have read and agreed to the published version of the manuscript.

## CONFLICT OF INTEREST STATEMENT

The Authors did not report any conflict of interest.

## Supporting information


**Supplemental Figure S1.** LBD40 transcript does not overaccumulate in 35S:LBD40 ECT compared with developing Col wt seeds (7–8 daf). NS, not significant.
**Supplemental Table S1.** Oligonucleotides used in this study. All oligonucleotides are written 5′ to 3′.
**Supplemental Table S2:** genes potentially directly expressed by LBD40 (Dataset S4) for which data supports direct expression by AGL15.
**Supplemental Table S3:** genes potentially directly repressed by LBD40 (Dataset S4) for which data supports direct repression by AGL15.

**Dataset S1:** LBD40 bound potential targets common in 3 reps (2/3 method).

**Dataset S2.** Repressed Targets for lbd40/41:: Col, SAMSE (10 days).
Dataset S2. Expressed Targets for lbd40/41:: Col, SAMSE (10 days).

**Dataset S3.** Repressed Targets for lbd40/41:: Col, seeds (7‐8daf ).
**Dataset S3.** Expressed Targets for lbd40/41:: Col, seeds (7‐8daf ).

**Dataset S4:** For SAM SE.
Dataset S4: For seeds.

**Dataset S5:** Number of genes responsive to LBD40 accumulation in SAM SE and seeds, including potential direct (Bound) and indirect targets.

**Dataset S6:** common potential bound targets of LBD40 and AGL15.
