# Supplementary material for: Regulatory mechanisms of the LBD40 transcription factor in Arabidopsis thaliana somatic embryogenesis
Source: Plant Direct. 2023 Dec 6;7(12):e547. doi: 10.1002/pld3.547 (PMC10699890; doi:10.1002/pld3.547)
Supplement: Supplementary file 1 — Supplemental Figure S1. LBD40 transcript does not overaccumulate in 35S:LBD40 ECT compared with developing Col wt seeds (7–8 daf). NS, not significant. Supplemental Table S1. Oligonucleotides used in this study. All oligonucleotides are written 5′ to 3′. Supplemental Table S2: genes potentially directly expressed by LBD40 (Dataset S4) for which data supports direct expression by AGL15. Supplemental Table S3: genes potentially directly repressed by LBD40 (Dataset S4) for which data supports direct repression by AGL15. [file PLD3-7-e547-s001.pdf]

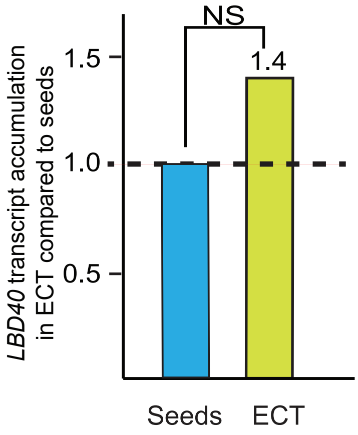

Supplemental Figure S1.
LBD40 transcript does not overaccumulate in *35S:LBD40* ECT tissue compared to developing Col wt seeds (7-8 daf). NS, not significant.

Supplemental Table S1. Oligonucleotides used in this study. All oligonucleotides are written 5’ to 3’.

**AGI**	**Gene name**	**Forward**	**Reverse**
**For cloning**			
AT1G67100	LOB DOMAIN-CONTAINING PROTEIN 40 (LBD40)	CACCATGCGTATGAGTTGTAACGGATG	CTCACTGCACACCAGTCCAAGCTC
**For ChIP-qPCR**			
Non-bound region (NB1)	INTERGENIC REGION	GAACTACTCGGTTTGCGAATTG	CCTTGCCGATCCTGATGAATA
AT5G64310	ARABINOGALACTAN PROTEIN 1 (AGP1)	TCTCCCATTCGTTTGCTGAA	CTACTACGCAAGTCTCTCCTCTA
AT1G01920	SET DOMAIN-CONTAINING PROTEIN (SET)	ATATCGATCGAGGCTGTTTA	CGGTGTTAGGAAAATCAATC
AT4G02380	SENESCENCE-ASSOCIATED GENE 21 (LEA5)	GTGTCTCTTCCTCTCTT	GGGAAGTTGTAGCTTTG
AT5G57660	CONSTANS-LIKE 5 (COL5)	GGATTCGGCTTAGAGAGTATC	TGAGTCAACTCGACAGAACA
AT4G32551	LISH DIMERISATION MOTIF;WD40/YVTN REPEAT-LIKE-CONTAINING DOMAIN (LUG)	CATCATCATCAGCAACAG	GAGAAGGTGGTTGGTTT
AT5G24470	PSEUDO-RESPONSE REGULATOR 5 (PRR5)	TCTCTCTTCCTCCGAACAGATT	GGATTAGCGAAGCGTTGAGTAG

AGI	Gene name	Forward	Reverse
AT2G40220	ABA INSENSITIVE 4 (ABI4)	GGTCCCACCAT TAAGGAAGATG	TCCTCCACTCAA ACCCTAAGA
**For qRT-PCR**			
AT5G19770	*TUBULIN ALPHA-3 (TUA3)*	TGGTGCCCAAC TGGGTTCAAAT	ACCTCTGCAACT GCTGTGTTGT
AT1G67100	*LOB DOMAIN-CONTAINING PROTEIN 40 (LBD40)*	TTGTTGTACGA AGCATGCGGGA	AGATGCCAGTT TCCTGACCACA
AT3G02550	*LOB DOMAIN-CONTAINING PROTEIN 41 (LBD41)*	CTATGGTTCGG TGGGTTTGT	CGATCTCTTTGA CCGGTTCTC
AT5G64310	ARABINOGALACTAN PROTEIN 1 (AGP1)	CATCTGCGATC TCCGATTCTC	TAACATAACCG CCACAGATCC
AT1G01920	*SET DOMAIN-CONTAINING PROTEIN (SET)*	CCATGAAGAAT CGAG GGACTT	CAGTCTTGTGA GCTGCTTCT
AT4G02380	*SENESCENCE-ASSOCIATED GENE 21 (LEA5)*	CCAGAAGATTT CTTG GGTTCCA	CTCAGCCGCGT CAATCTC
AT5G57660	*CONSTANS-LIKE 5 (COL5)*	TCGTTACGCTT CGAG GAAAG	GCTGAAGCATA AACA TGACTAAGG
AT5G24470	PSEUDO-RESPONSE REGULATOR 5 (PRR5)	AGAGATCCCAA CACAGCTTATA C	GAATGGGTGAA ACATGGAACTG

Supplemental Table S2: genes potentially directly expressed by LBD40 (Dataset S4) for which data supports direct expression by AGL15.

Gene ID	Gene Description	AGL15 bound as determined by ChIP-chip and/or ChIP-seq	Expressed (Exp) in response to AGL15 accumulation
AT1G05270	TraB family protein	Y (1,2)	EXP (3)
AT1G51500	ABC-2 type transporter family protein	Y (2)	EXP (4)
AT1G64640	early nodulin-like protein 8	Y (2)	EXP (4)
AT2G01010	rRNA	Y (2)	EXP (4)
AT2G01020	rRNA	Y (2)	EXP (5)
AT2G33210	heat shock protein 60-2	Y (2)	EXP (4)
AT3G27770	HUP53, HYPOXIA RESPONSE UNKNOWN PROTEIN 53	Y (2)	EXP (4)
AT4G33150	lysine-ketoglutarate reductase/saccharopine dehydrogenase	Y (1,2)	EXP (3,4)
AT5G24470	pseudo-response regulator 5	Y (1,2)	EXP (3,4,5)
AT5G49970	pyridoxin (pyrodoxamine) 5'-phosphate oxidase	Y (2)	EXP (4)
AT5G53170	FTSH protease 11	Y (2)	EXP (3,4)

Binding sites:

(1) - data presented in Zheng et al., (2009) using ChIP-chip to determine AGL15 binding sites and expression microarrays to determine response to AGL15 accumulation

(2) - data presented in Paul et al., (2022) using ChIP-seq to determine AGL15 binding sites

Response of gene expression:

(3) - data presented in Zheng et al., (2009) to determine significant transcript abundance of direct AGL15 targets in response to AGL15 accumulation (*agl15/18* compared to Col, wt and/or *35S:AGL15* compared to Col, wt) using microarrays.

(4) - data presented in Paul et al., (2022) using RNA-seq to determine significant transcript abundance of direct AGL15 targets to AGL15 accumulation (*agl15/18* compared to Col, wt).

(5) - data presented in Joshi et al., (2022) using RNA-seq to determine significant transcript abundance of direct AGL15 targets to AGL15 accumulation (*35S:AGL15* compared to Col, wt).

Supplemental Table S3: genes potentially directly repressed by LBD40 (Dataset S4) for which data supports direct repression by AGL15.

Gene ID	Gene Description	AGL15 bound as determined by ChIP-chip and/or ChIP-seq	Repressed (REP) in response to AGL15 accumulation
AT1G07720	3-ketoacyl-CoA synthase 3	Y(2)	REP (3)
AT1G18580	galacturonosyltransferase 11	Y(2)	REP (4)
AT1G28050	B-box type zinc finger protein with CCT domain	Y(2)	REP (4,5)
AT1G31350	KAR-UP F-box 1	Y(1,2)	REP (3)
AT2G28630	3-ketoacyl-CoA synthase 12	Y(2)	REP (4,5)
AT2G35930	plant U-box 23	Y(2)	REP (4,5)
AT2G37590	DNA binding with one finger 2.4	Y(2)	REP (3)
AT2G39020	Acyl-CoA N-acyltransferases (NAT) superfamily protein	Y(2)	REP (3,4,5)
AT2G40220	Integrase-type DNA-binding superfamily protein, ABI4	Y(2)	REP (3,4)
AT2G43020	polyamine oxidase 2	Y(2)	REP (5)
AT2G43130	P-loop containing nucleoside triphosphate hydrolases superfam	Y(2)	REP (4,5)
AT2G43800	Actin-binding FH2 (formin homology 2) family protein	Y(2)	REP (5)
AT2G47500	P-loop nucleoside triphosphate hydrolases superfamily protein	Y(2)	REP (4,5)
AT3G28340	galacturonosyltransferase-like 10	Y(1,2)	REP (3,4,5)
AT3G28345	ABC transporter family protein	Y(1,2)	REP (4,5)
AT3G28920	homeobox protein 34	Y(2)	REP (3)
AT3G55950	CRINKLY4 related 3	Y(2)	REP (3)
AT3G60080	RING/U-box superfamily protein	Y(1)	REP (3)
AT4G13710	Pectin lyase-like superfamily protein	Y(2)	REP (4,5)
AT4G27657	Hypothetical protein	Y(1,2)	REP (3,4)
AT5G02750	RING/U-box superfamily protein	Y(2)	REP (4,5)
AT5G09470	dicarboxylate carrier 3	Y(2)	REP (3)
AT5G13290	Protein kinase superfamily protein	Y(2)	REP (4)
AT5G59960	K-stimulated pyrophosphate-energized sodium pump protein	Y(2)	REP (4)
AT5G64310	arabinogalactan protein 1	Y(1)	REP (3)

Binding sites:

(1) - data presented in Zheng et al., (2009) using ChIP-chip to determine AGL15 binding sites and expression microarrays to determine response to AGL15 accumulation

(2) - data presented in Paul et al., (2022) using ChIP-seq to determine AGL15 binding sites

Response of gene expression:

(3) - data presented in Zheng et al., (2009) to determine significant transcript abundance of direct AGL15 targets in response to AGL15 accumulation (*agl15/18* compared to Col, wt and/or *35S:AGL15* compared to Col, wt) using microarrays.

(4) - data presented in Paul et al., (2022) using RNA-seq to determine significant transcript abundance of direct AGL15 targets to AGL15 accumulation (*agl15/18* compared to Col, wt).

(5) - data presented in Joshi et al., (2022) using RNA-seq to determine significant transcript abundance of direct AGL15 targets to AGL15 accumulation (*35S:AGL15* compared to Col, wt).
